# Survival from cancer in teenagers and young adults in England, 1979–2003

**DOI:** 10.1038/sj.bjc.6604460

**Published:** 2008-08-19

**Authors:** J M Birch, D Pang, R D Alston, S Rowan, M Geraci, A Moran, T O B Eden

**Affiliations:** 1 Cancer Research UK Paediatric and Familial Cancer Research Group, University of Manchester, Royal Manchester Children's Hospital, Stancliffe, Hospital Road, Manchester M27 4HA, UK; 2 National Cancer Intelligence Centre, Office for National Statistics, 1 Drummond Gate, London SW1V 2QQ, UK; 3 North West Cancer Intelligence Service, Christie Hospital, Withington, Manchester M20 4BX, UK; 4 Academic Unit of Paediatric and Adolescent Oncology, University of Manchester, Teenage Cancer Trust Young Oncology Unit, Christie Hospital NHS Trust, Withington, Manchester M20 4BX, UK

**Keywords:** cancer survival, trends, Teenagers and Young Adults, national cancer statistics, socioeconomic deprivation

## Abstract

Cancer is the leading cause of disease-related death in teenagers and young adults aged 13–24 years (TYAs) in England. We have analysed national 5-year relative survival among more than 30 000 incident cancer cases in TYAs. For cancer overall, 5-year survival improved from 63% in 1979–84 to 74% during 1996–2001 (*P*<0.001). However, there were no sustained improvements in survival over time among high-grade brain tumours and bone and soft tissue sarcomas. Survival patterns varied by age group (13–16, 17–20, 21–24 years), sex and diagnosis. Survival from leukaemia and brain tumours was better in the youngest age group but in the oldest from germ-cell tumours (GCTs). For lymphomas, bone and soft tissue sarcomas, melanoma and carcinomas, survival was not significantly associated with age. Females had a better survival than males except for GCTs. Most groups showed no association between survival and socioeconomic deprivation, but for leukaemias, head and neck carcinoma and colorectal carcinoma, survival was significantly poorer with increasing deprivation. These results will aid the development of national specialised service provision for this age group and identify areas of clinical need that present the greatest challenges.

In 2004 in England, over 50% of cancers were diagnosed at age 70 years and above, with only 0.5% in 15–24-year-olds (Office for National Statistics, 2006). However, cancer is the leading disease-related cause of death in this age range (Geraci *et al*, 2007). There is growing recognition that young cancer patients have special physical, social and educational needs in addition to appropriate disease-specific treatment. Risks of developing treatment-induced second malignancies and organ dysfunction are critical considerations in the young. Loss of fertility and other organ-specific cytotoxic effects and disruption to education, vocational and professional training can have a profound influence on future life (National Collaborating Centre for Cancer, 2005).

Detailed national survival data for this age group have not been reported hitherto but are important for service planning and as a baseline for monitoring progress. We now describe survival trends over time and patterns of survival by age, sex and socioeconomic deprivation for a 23-year series of 13- to 24-year-olds with cancer in England.

## Materials and methods

Teenagers and young adults aged 13–24 years (TYAs) diagnosed with malignancy in England, during the period 1979–2001, followed up to 31 December 2003, were included in this study. National cancer registration data on individual eligible cases were supplied by the National Cancer Intelligence Centre, Office for National Statistics, London (ONS), including dates of birth, diagnosis and follow-up, sex, histological type and primary site of cancer, Townsend deprivation index score (TDI) and vital status. Cases with vital status unknown (patient record not traced at the National Health Service Central Register) were excluded as were cases with a survival time of zero (diagnosed and died on the same day). These exclusion criteria were those applied by Coleman *et al* (1999). Cases lost to follow-up, for example, emigrated, were included up to date last known to be alive.

For cases registered from 1979 to 1994, cancer morphology was coded according to the International Classification of Diseases for Oncology, first edition (ICD-O 1) (World Health Organization, 1976), and cancer site according to the ninth revision of the International Classification of Diseases (ICD 9) (World Health Organization, 1977). For cases registered during the period 1995–2001, diagnoses were similarly coded according to ICD-O, second edition (ICD-O 2) (Percy *et al*, 1990), and ICD tenth revision (ICD-10) (World Health Organization, 1992). The cases were classified into 10 main diagnostic groups and 2–7 subgroups per main group as described previously (Birch *et al*, 2002). This classification is recognised internationally as a suitable vehicle for analysing data on TYAs (Barr *et al*, 2006).

We examined survival for each diagnostic subgroup by age at diagnosis (13–16, 17–20, 21–24 years), TDI, sex and calendar period (1979–1984, 1985–1989, 1990–1995, 1996–2001). Cases were divided into five groups, from the most affluent to most deprived, on the basis of the quintile of the distribution of TDIs for census ward of residence. TDIs are derived from levels of four census variables: car ownership; house ownership; overcrowding and unemployment within wards, giving a measure of material deprivation (Townsend *et al*, 1998).

Five-year relative survival in each diagnostic group was calculated by dividing observed by expected survival among comparable groups in the general population (Ederer *et al*, 1961). The 5-year expected survival was derived from the age, sex, deprivation and calendar year-specific national mortality rates for England (Coleman *et al*, 1999). Relative survival by age, sex, calendar period and TDI was examined using Poisson regression (Dickman *et al*, 2004, Breslow and Day, 1987). Specified diagnostic subgroups with 250 (0.8%) or more cases as well as smaller groups of interest were examined individually. The significance level was set at 5%. Analyses were carried out using the statistical software package Stata, Version 9 (StataCorp LP, 2005).

## Results

Survival time was available for 31 876 (94.8%) of 33 625 potentially eligible patients. During the period from 1993 (when such information became available) to 2001, 142 of 217 (65.4%) with zero survival time were registered by death certificate only. The remaining cases were hospital registrations who were diagnosed and died on the same day.

**Figure 1:**
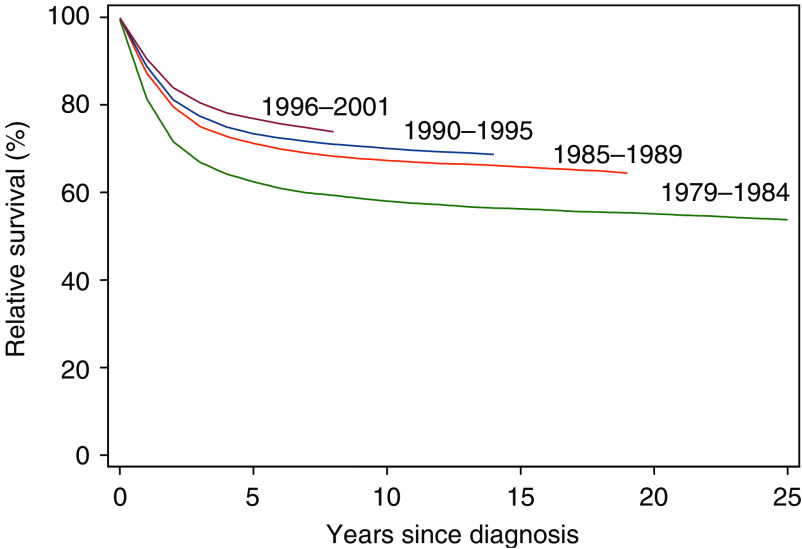
Relative survival of cancer patients aged 13–24 years, diagnosed between 1979 and 2001 in England, by calendar period.

Figure 1 shows that overall 5-year relative survival steadily increased throughout the study period (*P*<0.001) from 63% in 1979–1984 to 77% in 1996–2001. The most marked increase was between the two earliest periods.

Table 1 shows that 5-year relative survival was significantly better for females than males except for germ cell tumours (GCTs) and central nervous system (CNS) tumours. The pattern of survival with age varied between diagnostic groups. For GCTs in 13- to 16-years-olds, 17- to 20-year-olds and 21- to 24-year-olds, survival was 80, 87 and 90% respectively. However, for leukaemia and CNS tumours, survival was better in the youngest group (*P*<0.001). For lymphomas, bone sarcomas, soft tissue sarcomas (STSs), melanoma and carcinomas, survival was not significantly associated with age. For leukaemia and carcinomas, the most deprived groups had the lowest survival (*P*=0.048 and 0.008, respectively). All diagnostic groups showed improvements in 5-year relative survival during the study period of between 9% (CNS) and 21% (leukaemia), except STS for which no improvement was seen.

Tables 2, 3, 4, 5 and 6 present the results of analyses by the major subtypes within main diagnostic groups.

Table 2 shows 5-year survival of patients with haematological malignancies. For acute lymphoid leukaemia (ALL), females had a better survival than males (50 *vs* 43%, *P*=0.019), survival decreased with increasing age (*P*<0.001) and increased by 18% during the study period from 37 to 55% (*P*<0.001). For acute myeloid leukaemia (AML), there was an even greater improvement over time, from 27 to 50% but from a lower starting point than ALL. There were no significant differences in survival from AML by age and sex. For chronic myeloid leukaemia, there was a marked improvement in survival between the time periods 1979–1984 and 1985–1989 but no consistent further improvement. Although there was a significant trend with TDI for leukaemia overall (Table 1) there was no significant trend for any subtype of leukaemia.

For non-Hodgkin lymphoma (NHL), 13- to 16-year-olds had a better survival than 17- to 24-year-olds (*P*=0.033). There was a marked improvement in survival between 1979–84 and 1985–89 but little subsequent improvement. For Hodgkin lymphoma (HL), female patients had a small survival advantage over males (*P*=0.026), with significant improvements over time (*P*<0.001), reaching 93% in the latest period. There was no significant trend in survival with TDI for either NHL or HL.

Table 3 shows 5-year relative survival of patients with selected CNS tumours. Only astrocytomas showed decreased survival with increasing age (*P*<0.001), and this group showed no improvement over the study period. This pattern was driven by high-grade astrocytoma (HGA), which is more common in older age groups (data not shown). For HGA, 5-year survival did not improve during the study period (*P*=0.85) and a very low survival rate of 14% was seen in the latest period. However, for low-grade astrocytoma, 5-year survival improved from 76% in 1979–1984 to 90% in 1990–1995 but with no further improvement (*P*=0.005). The ‘other glioma’ group, mainly oligodendroglioma and ependymoma, showed marked improvements from 1979 to 1995 but no subsequent improvement. For medulloblastoma and supratentorial primitive neuroectodermal tumours, there were no significant differences in survival by sex, age and time period of diagnosis. No CNS tumour group showed a trend with TDI.

Table 4 shows 5-year relative survivals of patients with bone tumours and STS. For osteosarcoma, females had a better prognosis than males (*P*=0.008) and survival improved significantly between 1979–84 and 1985–89 but with no improvements more recently. Thirteen- to sixteen-year-olds with Ewing sarcoma did better than 17- to 24-year-olds (*P*=0.021), with a significant improvement in survival over time (*P*=0.005), again particularly marked between 1979–1984 and 1985–1989. Rhabdomyosarcoma (RMS) survival decreased with increasing age (*P*<0.001), with no improvement over time and 5-year survival of only 29% in the latest period. For other specified types of STS combined, there was no significant improvement in survival over time. For unspecified STSs, survival improved significantly between 1979 and 1995 but not subsequently. There were no significant trends in survival with TDI for any specified types of bone and STS.

Table 5 shows 5-year relative survivals among patients with GCTs by primary site. For testicular GCTs, survival increased with advancing age at diagnosis (*P*<0.001). There was a consistent improvement in 5-year survival over time (*P*<0.001), reaching 96% in the latest period. Ovarian GCTs showed similar patterns of survival. For CNS GCTs, there were no significant differences in survival by sex, age and period of diagnosis. For GCTs of other sites, females had a substantially superior 5-year survival (*P*<0.001). None of the subgroups showed a trend with TDI.

Table 6 shows 5-year relative survivals of patients with carcinomas of selected sites. For head and neck carcinomas (excluding thyroid), females had a better survival than males (*P*=0.001). There was a trend of decreasing survival with increasing deprivation, but no trend with age. Survival steadily improved over time (*P*=0.003). Thyroid carcinoma showed a 97% or higher survival throughout the study period, with no significant variations in age, sex and deprivation (data not shown). For carcinoma of lung, there was a significant improvement over time (*P*=0.046) and females had a higher survival (*P*=0.052). For carcinoma of breast, survival decreased with increasing age but the number of cases below age 21 years was very small. There was a borderline significant trend for improved survival over time (*P*=0.055). For ovarian carcinoma, there was a marked improvement in survival between 1990–1995 and 1996–2001. This may in part reflect changes in coding between ICD-O1 and ICD-O2 so that additional, lower-grade ovarian tumours were included in the 1996–2001 data. No trend with age was seen. Carcinoma of cervix showed a marked improvement between 1979–84 and 1985–89 (*P*=0.012), but survival has remained at the same level since. For colorectal carcinomas, females had a substantially better survival than males (*P*<0.001). Survival increased significantly over time, particularly in the most recent period. The most deprived group had the lowest survival (*P*=0.001). Numbers of carcinomas of other sites were too small for separate analysis.

## Discussion

This study presents the first national data for England on cancer survival among TYAs. It is now acknowledged that the special needs of cancer patients aged 0–18 years would be best served by principal treatment centres providing age-appropriate facilities and managed by dedicated multidisciplinary teams (National Collaborating Centre for Cancer, 2005). For 19- to 24-year-olds, unhindered access to such expert teams is recommended. It has been suggested that in this age group in the United States, low recruitment to clinical trials contributes to the comparatively poor improvements in cancer survival (Bleyer *et al*, 2007). A recent study has compared clinical trial inclusion rates of children with those of TYAs in Great Britain who have cancers relevant to both age ranges and for which phase III trials are in progress (leukaemia, lymphoma, CNS tumours, sarcoma and testicular GCT). Results for cases diagnosed in 2005–2007 show that 56% of total incident cases aged 5–14 years are entered into trials compared with only 20% of 15- to 24-year-olds. Trial inclusion for CNS tumours was particularly low (Whelan and Fern, 2008). The baseline data on survival trends presented here are of importance in monitoring future progress in cancer survival in TYAs and assessing the impact of new specialist TYA cancer units, including recruitment to clinical trials.

In this study, we have analysed survival by predefined morphological groups of cancers, appropriate to the TYA age range, using national data covering more than 30 000 incident cases over a 23-year period. Overall, there were marked improvements in survival during the study period especially for all subgroups of leukaemia and NHL. However, for certain other groups, the results are less encouraging. Both osteosarcoma and Ewing sarcoma showed a step change in survival between the two early periods but then no further increase after 1989. There was no improvement for RMS and other STS. Although survival among children diagnosed with osteosarcoma up to 1997 in Britain was only slightly better than that in TYAs reported here, children with Ewing sarcoma and RMS showed more marked improvements and better survivals than their TYA counterparts (Stiller *et al*, 2006; Pastore *et al*, 2006). During 1993–1997, in children 5-year survival was 67% for Ewing sarcoma and 65% for RMS, but in TYAs during 1996–2001, the respective survivals were 42 and 29%. For RMS, this may partly be due to a higher proportion of TYA patients with more aggressive histologies, but this cannot entirely explain the poor outcomes in TYAs with bone and soft tissue sarcomas in general.

High-grade CNS tumours in TYAs showed little or no improvements in survival during the study period, and this was also the case for British children up to 1997 with comparable tumours (Peris-Bonet *et al*, 2006). Clearly, the clinical management of high-grade CNS tumours in young people presents a major challenge. In contrast, these results show consistently high survival rates for GCTs, with equivalent improvement in 5-year survival for both testicular and ovarian tumours. Across all ages, nearly all testicular tumours are of germ-cell origin, so that the high survival rates previously reported for testicular cancer can be interpreted as survival from GCTs of the testis. However, most ovarian cancers are carcinomas, and survival rates for ovarian cancer (Coleman *et al*, 1999) do not reflect survival from ovarian GCTs. This is particularly important in TYAs, as GCTs are the predominant ovarian malignancy in this age group (Birch *et al*, 2003).

About 80% of cancers overall are carcinomas, but these constitute only 16% of TYA cancers (Alston *et al*, 2007). Carcinomas of most sites in the present series show favourable survival rates compared with cancers at those sites across all ages (Coleman *et al*, 1999). This suggests that the TYA cases may have histologically lower-grade tumours than older cases and/or differ biologically. However, survival from breast carcinoma in TYAs is similar to breast cancer in general (Coleman *et al*, 1999). Females have higher survival rates for most types of cancer, and this holds true for TYAs; possible explanations include earlier presentation in females and less aggressive tumour biology.

Cancer survival rates among older patients in England and Wales are strongly influenced by socioeconomic status (Coleman *et al*, 1999), but only small non-significant differences were seen in children. In this study, most cancers showed no association between TDI and survival, but for leukaemias and carcinomas overall, there were significant trends towards poorer survival with increasing deprivation, particularly for colorectal and head and neck carcinomas. These latter cancers, which show marked associations between survival and deprivation in older patients (Coleman *et al*, 1999), are aetiologically linked to lifestyle factors also associated with deprivation, such as tobacco smoking and poor diet (IARC, 1986; Key *et al*, 2004). These factors may influence survival due to general poor health. Other considerations include speed of seeking medical healthcare, referral patterns and clinical management in socioeconomically deprived areas.

In conclusion, although there were marked increases in survival over time for all cancers combined, for some diagnostic groups, little or no improvements were seen. These results provide baseline data against which to compare outcomes in patients treated in the developing specialist TYA cancer units and in those entered into clinical trials. The data serve to identify the patient groups that present the greatest clinical challenges.

## Figures and Tables

**Table 1:** Five-year relative survival (%) of patients diagnosed at age 13–24 during 1979–2001 in England by main diagnostic group

	**All**			**Leukaemia**			**Lymphoma**			**CNS**			**Bone sarcomas**			**STS**			**GCTs**			**Melanoma**			**Carcinomas**		
	***N***	**%**	***P***	***N***	**%**	***P***	***N***	**%**	***P***	***N***	**%**	***P***	***N***	**%**	***P***	***N***	**%**	***P***	***N***	**%**	***P***	***N***	**%**	***P***	***N***	**%**	***P***
*Sex*																											
Male	17 339	69	<0.001	2129	42	0.016	4607	80	<0.001	1777	59	0.122	1170	46	0.019	950	52	0.012	4022	89	0.730	928	78	<0.001	1548	63	<0.001
Female	14 433	73		1393	47		3583	84		1382	62		769	52		793	58		520	86		1768	89		3931	76	
																											
*Age (years)*																											
13–16	6682	65	<0.001	1303	47	<0.001	1644	82	0.772	1092	68	<0.001	815	48	0.611	495	58	0.455	381	80	<0.001	244	89	0.158	600	70	0.920
17–20	10 107	70		1194	42		2948	81		922	61		716	46		611	51		1386	87		786	84		1363	73	
21–24	14 983	74		1025	40		3598	82		1145	53		408	54		637	55		2775	90		1666	85		3516	72	
																											
*Deprivation*																											
Most affluent	6353	71	0.001	700	45	0.048	1705	83	0.073	653	61	0.914	408	50	0.904	346	51	0.844	916	87	0.212	595	86	0.861	928	73	0.008
2	6353	71		725	46		1635	82		661	60		412	48		320	55		919	89		584	85		1011	73	
3	6355	72		668	42		1663	82		648	62		366	47		339	60		914	89		571	85		1101	73	
4	6355	71		723	43		1570	82		599	60		394	48		384	53		884	89		514	83		1168	73	
Most deprived	6356	70		706	41		1617	80		598	60		359	51		354	53		909	89		432	87		1271	69	
																											
*Year of diagnosis*																											
1979–1984	7509	62	<0.001	935	33	<0.001	2031	77	<0.001	763	54	<0.001	544	39	<0.001	424	53	0.152	924	80	<0.001	454	74	<0.001	1309	64	<0.001
1985–1989	7482	71		789	43		2064	82		718	61		446	55		426	53		1063	85		606	86		1224	71	
1990–1995	8488	73		929	47		2121	83		869	64		468	52		447	55		1220	92		850	86		1432	74	
1996–2001	8293	77		869	54		1974	86		809	63		481	51		446	56		1335	94		786	90		1514	78	

CNS=central nervous system tumours; GCTs=germ-cell tumours; STS=soft tissue sarcoma.

**Table 2:** Five-year relative survival (%) of patients with haematological malignancies^a^ diagnosed at age 13–24 during 1979–2001 in England

	**ALL**			**AML**			**CML**			**NHL**			**HL**		
	***N***	**%**	***P***	***N***	**%**	***P***	***N***	**%**	***P***	***N***	**%**	***P***	***N***	**%**	***P***
*Sex*															
Male	1163	43	0.019	671	36	0.074	178	50	0.690	1552	65	0.206	3055	87	0.026
Female	633	50		565	42		116	54		789	68		2794	89	
															
*Age (years)*															
13–16	869	50	<0.001	333	40	0.536	54	43	0.372	599	70	0.033	1045	89	0.550
17–20	581	44		439	39		94	54		816	65		2132	88	
21–24	346	37		464	38		146	54		926	66		2672	88	
															
*Deprivation*															
Most affluent	361	48	0.066	255	39	0.253	45	73	0.173	457	62	0.813	1248	90	0.190
2	400	48		240	43		53	43		452	69		1183	86	
3	313	48		260	35		52	47		453	69		1210	86	
4	371	43		252	41		62	51		457	66		1113	89	
Most deprived	351	42		229	36		82	50		522	65		1095	87	
															
*Year of diagnosis*															
1979–1984	490	37	<0.001	311	27	<0.001	64	30	0.002	506	52	<0.001	1525	85	<0.001
1985–1989	416	45		259	34		71	62		569	70		1495	86	
1990–1995	457	48		351	43		77	51		629	68		1492	89	
1996–2001	433	55		315	50		82	66		637	72		1337	93	

ALL=acute lymphoid leukaemia; AML=acute myeloid leukaemia; CML=chronic myeloid leukaemia; HL=Hodgkin lymphoma; NHL=non-Hodgkin lymphoma.

a Excluding 196 cases with other rare and unspecified haematological malignancies.

**Table 3:** Five-year relative survival (%) of patients with selected CNS tumours diagnosed at age 13–24 during 1979–2001 in England

	**Astrocytoma**			**Other glioma**			**Ependymoma**			**PNET**			**Other specified and unspecified**		
	***N***	**%**	***P***	***N***	**%**	***P***	***N***	**%**	***P***	***N***	**%**	***P***	***N***	**%**	***P***
*Sex*															
Male	869	60	0.439	376	53	0.125	141	76	0.291	191	51	0.295	200	60	0.092
Female	749	59		251	61		110	84		117	57		155	69	
															
*Age (years)*															
13–16	574	71	<0.001	183	62	0.249	94	77	0.196	129	60	0.058	112	68	0.383
17–20	480	61		179	56		77	81		89	49		97	66	
21–24	564	47		265	53		80	82		90	49		146	59	
															
*Deprivation*															
Most affluent	335	59	0.830	129	59	0.921	52	83	0.246	62	53	0.750	75	64	0.623
2	328	60		123	57		54	85		74	55		82	54	
3	324	61		134	57		52	76		65	59		73	68	
4	312	61		131	49		44	78		48	50		64	67	
Most deprived	319	57		110	62		49	75		59	49		61	67	
															
*Year of diagnosis*															
1979–1984	359	56	0.199	184	49	0.002	64	71	0.014	78	50	0.118	78	49	0.006
1985–1989	347	62		153	55		62	74		66	46		90	69	
1990–1995	443	60		159	64		61	89		77	59		129	65	
1996–2001	469	59		131	61		64	85		87	61		58	74	

PNET=medulloblastoma and primitive neuroectodermal tumours.

**Table 4:** Five-year relative survival (%) of patients with bone and soft tissue sarcomas diagnosed at age 13–24 during 1979–2001 in England

	**Osteosarcoma**			**Ewing sarcoma**			**RMS**			**Other specified STS**			**Unspecified STS**		
	***N***	**%**	***P***	***N***	**%**	***P***	***N***	**%**	***P***	***N***	**%**	***P***	***N***	**%**	***P***
*Sex*															
Male	638	44	0.008	356	37	0.990	241	31	0.329	403	57	0.446	188	51	0.069
Female	407	53		215	39		161	38		397	61		133	58	
															
*Age (years)*															
13–16	480	47	0.810	247	44	0.021	172	42	<0.001	191	67	0.065	80	59	0.159
17–20	394	48		207	30		159	30		276	56		112	55	
21–24	171	48		117	38		71	25		333	56		129	51	
															
*Deprivation*															
Most affluent	216	48	0.996	118	42	0.974	92	33	0.733	157	58	0.258	62	50	0.063
2	216	46		127	35		71	41		149	63		58	45	
3	198	46		117	37		76	34		148	61		68	62	
4	215	48		114	34		82	28		177	57		77	56	
Most deprived	200	48		95	41		81	36		169	55		56	57	
															
*Year of diagnosis*															
1979–1984	311	38	<0.001	152	26	0.005	92	33	0.430	153	58	0.294	71	44	0.007
1985–1989	235	55		124	41		122	39		168	56		77	49	
1990–1995	246	50		138	43		99	33		217	57		98	61	
1996–2001	253	49		157	42		89	29		262	62		75	59	

RMS=rhabodomyosarcoma; STS=soft tissue sarcoma.

**Table 5:** Five-year relative survival (%) of patients with GCTs diagnosed at age 13–24 during 1979–2001 in England

	**Testis**			**Ovary**			**CNS**			**Others**		
	***N***	**%**	***P***	***N***	**%**	***P***	***N***	**%**	***P***	***N***	**%**	***P***
*Sex*												
Male	3788	90					132	73	0.260	102	51	<0.001
Female				417	87		26	81		77	85	
												
*Age (years)*												
13–16	155	83	<0.001	142	83	0.120	63	76	0.329	21	57	0.348
17–20	1120	89		145	87		54	80		67	64	
21–24	2513	92		130	90		41	66		91	68	
												
*Deprivation*												
Most affluent	755	89	0.267	97	82	0.716	35	77	0.413	29	45	0.246
2	775	90		72	90		37	81		35	71	
3	763	92		81	86		33	69		37	65	
4	742	91		84	89		22	78		36	69	
Most deprived	753	91		83	85		31	68		42	72	
												
*Year of diagnosis*												
1979–1984	734	83	<0.001	105	72	<0.001	29	66	0.178	56	63	0.083
1985–1989	908	87		86	85		28	64		41	59	
1990–1995	1010	93		109	94		57	83		44	71	
1996–2001	1136	96		117	94		44	77		38	71	

**Table 6:** Five-year relative survival (%) of patients with selected carcinoma diagnosed at age 13–24 during 1979–2001 in England

	**Head and neck^a^**			**Lung**			**Female breast**			**Ovary**			**Cervix**			**Colorectal**			**Other GU**			**Other GI**		
	***N***	**%**	***P***	***N***	**%**	***P***	***N***	**%**	***P***	***N***	**%**	***P***	***N***	**%**	***P***	***N***	**%**	***P***	***N***	**5 years**	***P***	***N***	**5 years**	***P***
*Sex*																								
Male	305	70	0.001	80	46	0.052										268	52	<0.001	312	83	0.002	177	25	0.956
Female	271	82		66	63		458	61		572	79		886	79		281	69		206	72		166	20	
																								
*Age (years)*																								
13–16	143	74	0.375	17	64	0.433	10	70	0.041	43	78	0.425	5	60	0.293	86	60	0.090	43	67	0.003	48	28	0.055
17–20	186	72		32	56		57	75		164	84		75	87		164	60		138	73		104	27	
21–24	247	80		97	51		391	58		365	76		806	78		299	62		337	82		191	19	
																								
*Deprivation*																								
Most affluent	102	82	0.034	24	57	0.109	77	57	0.985	111	72	0.258	103	82	0.703	108	66	0.001	99	81	0.299	53	24	0.842
2	111	75		23	65		104	62		101	85		142	76		100	71		93	77		61	26	
3	111	78		27	63		78	65		112	78		188	78		113	59		110	82		68	20	
4	122	76		35	59		104	56		117	76		206	81		98	65		104	79		82	21	
Most deprived	130	69		37	32		95	62		131	83		247	78		130	48		112	74		79	24	
																								
*Year of diagnosis*																								
1979–1984	146	67	0.003	39	46	0.046	103	53	0.055	119	75	0.013	199	69	0.012	127	54	0.003	132	73	0.091	93	19	<0.001
1985–1989	120	74		28	50		113	63		119	76		219	81		111	58		124	83		83	22	
1990–1995	137	78		40	50		127	62		138	73		243	84		124	58		160	78		83	19	
1996–2001	173	83		39	67		115	64		196	87		225	80		187	70		102	83		84	31	

a Excluding thyroid.
